# Change in organizational justice as a predictor of insomnia symptoms: longitudinal study analysing observational data as a non-randomized pseudo-trial

**DOI:** 10.1093/ije/dyw293

**Published:** 2017-01-08

**Authors:** Tea Lallukka, Jaana I Halonen, Børge Sivertsen, Jaana Pentti, Sari Stenholm, Marianna Virtanen, Paula Salo, Tuula Oksanen, Marko Elovainio, Jussi Vahtera, Mika Kivimäki

**Affiliations:** 1 Finnish Institute of Occupational Health, Helsinki, Turku & Kuopio, Finland; 2 Department of Public Health, Clinicum, University of Helsinki, Finland; 3 Department of Health Promotion, Norwegian Institute of Public Health, Bergen, Norway; 4 Regional Centre for Child and Youth Mental Health and Child Welfare, Uni Research Health, Bergen, Norway; 5 Department of Psychiatry, Helse Fonna HF, Haugesund, Norway; 6 Department of Public Health, University of Turku, Turku, Finland; 7 Department of Psychology, University of Turku, Turku, Finland; 8 Institute of Behavioural Sciences, University of Helsinki, Finland; 9 Department of Health and Social Care Systems, National Institute for Health and Welfare, Helsinki, Finland; 10 Turku University Hospital, Turku, Finland; 11 Department of Epidemiology and Public Health, University College London, London, UK

**Keywords:** Insomnia symptom, organizational justice, psychosocial, longitudinal, changes over time, employees

## Abstract

**Background:**

Despite injustice at the workplace being a potential source of sleep problems, longitudinal evidence remains scarce. We examined whether changes in perceived organizational justice predicted changes in insomnia symptoms.

**Methods:**

Data on 24 287 Finnish public sector employees (82% women), from three consecutive survey waves between 2000 and 2012, were treated as ‘pseudo-trials’. Thus, the analysis of unfavourable changes in organizational justice included participants without insomnia symptoms in Waves 1 and 2, with high organizational justice in Wave 1 and high or low justice in Wave 2 (*N* = 6307). In the analyses of favourable changes in justice, participants had insomnia symptoms in Waves 1 and 2, low justice in Wave 1 and high or low justice in Wave 2 (*N* = 2903). In both analyses, the outcome was insomnia symptoms in Wave 3. We used generalized estimating equation models to analyse the data.

**Results:**

After adjusting for social and health-related covariates in Wave 1, unfavourable changes in relational organizational justice (i.e. fairness of managerial behaviours) were associated with increased odds of developing insomnia symptoms [odds ratio = 1.15; 95% confidence interval (CI) 1.02-1.30]. A favourable change in relational organizational justice was associated with lower odds of persistent insomnia symptoms (odds ratio = 0.83; 95% CI 0.71-0.96). Changes in procedural justice (i.e. the fairness of decision-making procedures) were not associated with insomnia symptoms.

**Conclusions:**

These data suggest that changes in perceived relational justice may affect employees’ sleep quality. Decreases in the fairness of managerial behaviours were linked to increases in insomnia symptoms, whereas rises in fairness were associated with reduced insomnia symptoms.

**Key Messages**

- In the workplaces studied, decreases in the perceived fairness of managerial behaviours were linked to increases in insomnia symptoms among employees.
- In contrast, rises in the perceived fairness of managerial behaviours were associated with reduced persistence of insomnia symptoms.
- Changes in the perceived fairness of managerial procedures were not associated with insomnia symptoms.

## Introduction

Insomnia symptoms are common in working populations, and the evidence of an association with the increased risk of work disability emphasizes the need for identifying new targets for insomnia prevention.^1–3^ An adverse psychosocial environment at work, consisting of factors such as high perceived demands, low job control, and effort-reward imbalance, are linked to an increased riskof insomnia.^4^^,^^5^ Moreover, low organizational justice has recently been found to be associated with insomnia and disturbed sleep,^6–9^ although this is not a universal observation.^10^ Organizational justice is a multidimensional concept, which refers to the fairness of decision-making processes (the procedural component), how equally supervisors treat employees and share information and whether employees themselves perceive that their viewpoints are taken into account (relational component).^11–13^

Examining the effects of organizational justice in randomized trials is challenging due to ethical and logical reasons. One limitation in the conventional prospective analysis of observational data is that the timing of the exposure and the outcome is difficult to determine convincingly. However, observational data with repeated measurements of organizational justice and insomnia symptoms would allow the opportunity to mimic a trial.^14^ Analysing repeat data as pseudo-trials by using the clearly defined inclusion and exclusion criteria of the participants could address questions such as whether a decline in organizational justice is associated with increased odds of initially non-symptomatic employees with high organizational justice developing insomnia symptoms, and whether a favourable change in organizational justice is associated with lower odds of persistent insomnia symptoms among employees with initially low organizational justice and insomnia symptoms.

Accordingly, in this study of Finnish public sector employees, we used data from three repeated measures of organizational justice and insomnia symptoms to determine whether unfavourable changes in organizational justice were associated with an increase in new-onset insomnia symptoms among employees with no insomnia symptoms; and whether increases in organizational justice were linked to reductions in the persistence of symptoms among employees who initially had insomnia symptoms. To address the multidimensionality of the organizational justice concept,^12^ we examined the relational and procedural components separately.

## Methods

### Participants and study design

The data were from the ongoing Finnish Public Sector (FPS) study of employees from 10 municipalities and six hospital districts (21 hospitals) in Finland.^15^ The majority of these public sector employees are women (82%), and the cohort comprises a wide range of occupations. The first survey data were collected in 2000‒02 (*n* = 48 598 participants, response rate 68%), and continued similarly at 4-year intervals, i.e. in 2004 (*n* = 48 076, response rate 66%), 2008 (*n* = 52 891, response rate 71%) and 2012 (*n* = 53 133, response rate 69%).

**Figure 1.**
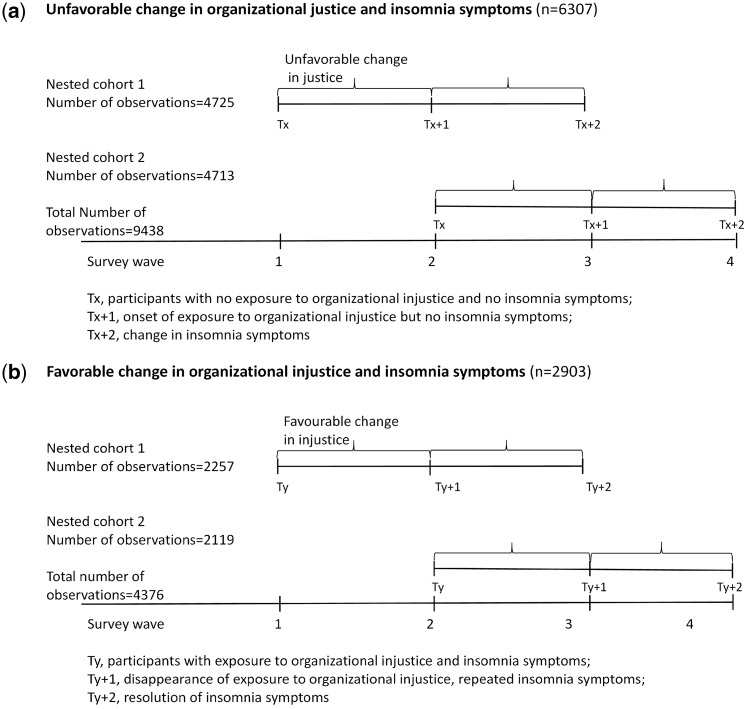
a. Setting of pseudo-trials used in analysis of unfavorable change in organizational justice. b. Setting of pseudo-trials used in analysis of favorable change in organizational injustice.

To maximize statistical power and the number of study members from the four data collection waves, we included two nested cohorts as ‘pseudo-trials’, based on the responses to the questions on organizational justice and insomnia symptoms in the three successive data waves (Figure 1a and b). The study design followed the principles of a clinical trial, with strict inclusion and exclusion criteria to address the questions regarding temporal order using observational data. To be included in these nested cohorts, a participant had to have participated in three consecutive survey waves, starting with either the 2000‒02 survey or the 2004 survey. For both nested cohorts, the first survey was considered the baseline (Wave 1). Altogether 24 287 participants (4431 men and 19 856 women) responded to three consecutive surveys from either the 2000‒02 baseline or the 2004 baseline onwards. Of these, 12 385 contributed to one of the nested cohorts, and 11 902 responded to all four surveys, contributing to both nested cohorts. This resulted in a total of 36 189 repeat observations. The differences in the baseline characteristics between the nested cohorts that began in 2000-02 and 2004 were small (online Appendix eTable 1, available as Supplementary data at *IJE* online).

For the analysis of unfavourable changes in organizational justice (Figure 1a), we included participants with high organizational justice in Wave 1, high or low organizational justice in Wave 2, and no insomnia symptoms in Waves 1 or 2 (*N* = 6307). Unfavourable changes in organizational justice refers to the group with high organizational justice in Wave 1 and low organizational justice in Wave 2, and the reference group comprises those who reported high organizational justice in Wave 1 and Wave 2. The outcome of interest was the presence or absence of insomnia symptoms in Wave 3.

In our analyses of favourable change in organizational justice (Figure 1b), we included participants with low organizational justice in Wave 1, high or low organizational justice in Wave 2, and the presence of insomnia symptoms in Waves 1 and 2 (*N* = 2903). A favourable change in organizational justice refers to participants with low organizational justice in Wave 1 and high organizational justice in Wave 2, and the reference group comprised participants with low organizational justice in Waves 1 and 2. The outcome of interest was the presence or absence of insomnia symptoms in Wave 3.

### Organizational justice

In Waves 1 and 2, the FPS questionnaires contained six statements on relational justice and seven statements on procedural justice.^11^^,^^12^ The statements assessing relational justice were: (i) your supervisor considers your viewpoint; (ii) your supervisor is able to suppress personal biases; (iii) you receive consistent information from line management (from your supervisor); (iv) your supervisor treats you with kindness and consideration; (v) your supervisor shows concern for your rights; (vi) your supervisor makes the effort to deal with you in an honest manner.

Procedural justice was assessed using the following statements: (i) procedures are designed to collect the accurate information necessary for making decisions; (ii) procedures are designed to provide opportunities to appeal or challenge a decision; (iii) procedures are designed to hear the concerns of all those affected by a decision; (iv) procedures are designed to generate standards so that decisions can be made with consistency; (v) procedures are designed to take the opinion of employees into account; (vi) procedures are designed to provide useful feedback; (vii) procedures are designed to provide clarification regarding decisions.

In each question, five response alternatives ranged from strongly disagree to strongly agree. The responses to the statements were summed, and the lowest tertile was used as a cut-off point to indicate low relational or procedural justice. This also allowed sufficient numbers in the analyses of change. All the others were categorized into the high organizational justice group. Cronbach’s alpha was 0.92 for the relational justice scale and 0.91 for the procedural justice scale.

### Insomnia symptoms

In Waves 1, 2 and 3, insomnia symptoms were measured using the Jenkins Sleep Problem scale covering difficulties initiating sleep, two questions about difficulties maintaining sleep, and non-restorative sleep.^16^ The respondent’s frequency of these four types of insomnia symptoms during the previous 4 weeks was elicited by providing six response alternatives; never, once a month, once a week, 2‒4 times a week, 5‒6 times per week, and nearly every night. All symptoms were combined to define ‘any insomnia symptoms’, i.e. participants who reported suffering from any of the four symptoms at least 2‒4 times per week. All the others belonged to the category of ‘no/rare insomnia symptoms’. The selected cut-off point was the closest to the diagnostic criteria of insomnia, with symptoms occurring at least three times per week.^17^ Changes in insomnia symptoms were examined as a change from the ‘no/rare insomnia symptoms’ category to having ‘any insomnia symptoms’, and from reporting ‘any insomnia symptoms’ to the category of ‘no/rare insomnia symptoms’.

### Covariates

In Wave 1, participants’ age and sex were derived from the registers of the employers, marital status from survey responses, and educational degree was based on the register from Statistics Finland (high = university degree, intermediate = high school or vocational school, low = comprehensive school). Shift work was self-reported (yes vs no). We also included self-reported low physical activity (metabolic equivalent, less than 14 MET-h per week),^18^^,^^19^ current smoking (yes vs no), heavy alcohol consumption (over 16 units/week for women and over 21 for men),^20^ body mass index (BMI, continuous in the models, kg/m^2^) and sleep apnoea (yes vs no). Physician-diagnosed asthma, chronic obstructive pulmonary disease, hypertension, diabetes and depression were combined into ‘comorbid conditions’ (no vs any comorbid disease).

### Ethical approval

The FPS study was approved by the ethics committees of the Finnish Institute of Occupational Health and the Hospital District of Helsinki and Uusimaa, Finland.

### Statistical methods

We fitted generalized estimating equation (GEE)-based models [odds ratios (OR) with 95% confidence intervals (CI)], allowing us to account for within-person correlation. This was done because participants could contribute to observations in two nested cohorts (‘pseudo-trials’). The analyses of change in organizational justice between Wave 1 and Wave 2 were adjusted for the covariates measured in Wave 1, in order to control for initial differences: (i) between participants who remained exposed to low organizational justice and those who experienced a change from low to high organizational justice; and (ii) between participants who remained exposed to high organizational justice and those who experienced a change from high to low organizational justice. Serial adjustments included Model 1, which was a crude model, adjusted for nested cohort (2000-02 vs 2004). Model 2 was further adjusted for sex, age, marital status, education, shift work, smoking, heavy alcohol consumption, low physical activity, BMI, sleep apnoea and comorbid conditions. As no sex interactions were found, men and women were analysed together. The analyses were conducted using SAS version 9.4.

## Results

The baseline characteristics of all the participants are displayed in Table 1, both in combination and separately for those included in the unfavourable (*n* = 6307) and favourable (*n* = 2903) change analyses. An unfavourable change occurred among 17.1% and a favourable change among 40.7% of the participants included in the trials. About a fifth of the participants were men, and the mean age was 43.5 [standard deviation (SD) 7.6 years] in the total study population, 42.8 (SD 7.6 years) among people in the unfavourable change analyses and 44.2 years (SD 7.1 years) among people in the favourable change analyses.

**Table 1.** Characteristics (numbers, %) of participants in analyses of unfavourable and favourable change in justice, and total study population: Finnish Public Sector Study

	Analysis of unfavourable change in justice between Waves 1 and 2 (*n* = 6307)		Analysis of favourable change in injustice between Waves 1 and 2 (*n* = 2903)		All participants (*n* = 24287)	
Sex	N	%	N	%	N	%
Men	1323	21.0	491	16.9	4431	18.2
Women	4984	79.0	2412	83.1	19856	81.8
Married						
Single	1310	20.9	739	25.8	5403	22.5
Living with a partner	4951	79.1	2124	74.2	18645	77.5
Education						
Low	501	7.9	246	8.5	2040	8.4
Intermediate	2183	34.6	949	32.7	8499	35.0
High	3623	57.4	1708	58.8	13748	56.6
Current smoker						
No	5100	82.7	2289	80.9	19518	82.5
Yes	1070	17.3	542	19.2	4137	17.5
Physically inactive						
No	4964	79.0	2136	73.8	18286	76.1
Yes	1318	21.0	758	26.2	5759	24.0
Heavy drinker						
No	5909	94.0	2562	88.7	22170	91.8
Yes	380	6.0	328	11.4	1989	8.2
Body mass index						
Normal weight	3708	59.6	1522	53.3	13573	57.2
Overweight	1896	30.5	943	33.0	7425	31.3
Obese	616	9.9	389	13.6	2734	11.5
Comorbid condition(s)						
No	5069	80.4	1738	59.9	17441	71.8
Yes	1238	19.6	1165	40.1	6846	28.2
Shift work						
No	4344	68.9	1841	63.4	16177	66.6
Yes	1963	31.1	1062	36.6	8110	33.4
Sleep apnoea						
No	6282	99.6	2847	98.1	24039	99.0
Yes	25	0.4	56	1.9	248	1.0

**Figure 2.**
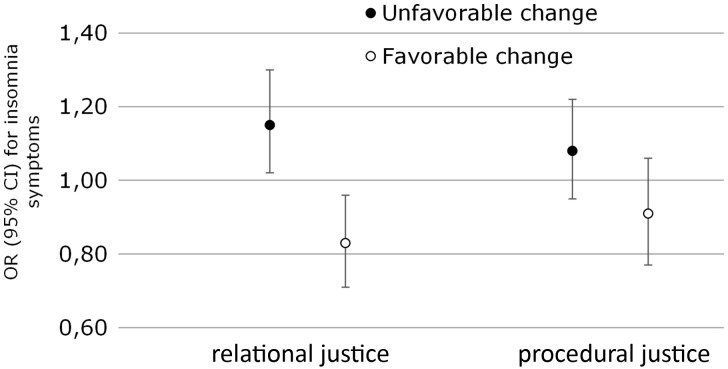
Associations between unfavorable change in relational and procedural justice and subsequent insomnia symptoms (n = 6307), and between a favorable change in relational and procedural injustice and repeated insomnia symptoms (n = 2903) among Finnish public sector employees. Adjusted for sociodemographic factors, shift work, and health-related covariates.

Among participants without insomnia symptoms, an unfavourable change in relational justice was associated with the onset of insomnia symptoms both in the crude analysis (OR 1.17; 95% CI 1.04-1.31), and after full adjustments (OR 1.15; 95% CI 1.02-1.30; Figure 2). However, no association was observed for unfavourable changes in procedural justice in the crude analysis (OR 1.08; 95% CI 0.95-1.23) or after adjustments (OR 1.08; 95% CI 0.95-1.22; Figure 2).

A favourable change in relational justice was associated with lower odds of repeated insomnia symptoms in the crude analysis (OR 0.83; 95% CI 0.71-0.96) and after adjustments (OR 0.83; 95% CI 0.71-0.96). However, we observed no beneficial effect on sleep of a corresponding favourable change in procedural justice in either the crude (OR 0.91; 95% CI 0.78-1.07) or the adjusted (OR 0.91; 95% CI 0.77-1.06) analyses.

The corresponding associations for overall organizational justice (sum of procedural and relational justice) reflected those observed for relational justice, although they were slightly weaker (see online Appendix eTable 2, available as Supplementary data at *IJE* online). Dropping depressive symptoms from the covariates of the fully adjusted model (online Appendix eTable 3a and b, available as Supplementary data at *IJE* online) or adding trait anxiety (in Wave 1) to covariates (online Appendix eTable 4a and b, available as Supplementary data at *IJE* online) had little effect on the results.

## Discussion

Our main finding was that the odds of new-onset insomnia symptoms increased by approximately 15% after a decrease in managerial fairness (i.e. relational justice), whereas rises in managerial fairness were associated with a 17% decrease in the odds of persistent symptoms. These associations were independent of the pertinent risk factors, including sociodemographic factors, health behaviours and comorbid conditions. We observed no association between changes in procedural justice and insomnia symptoms.

To our knowledge, this is the first study to examine the temporal association between a change in organizational justice and subsequent changes in insomnia symptoms, using observational data as a non-randomized pseudo-trial. This allowed us to better determine the direction of the associations than would cross-sectional or conventional prospective studies, which do not consider the temporal aspect or change in exposure. If the exposure and outcome are already present at baseline, or the outcome emerges over the follow-up between two time points, then it remains unclear whether the change in exposure predicts the change in the outcome.

There are few prospective studies on the association between organizational justice and sleep. A British study of white-collar civil servants found an association between repeated exposure to organizational injustice and sleeping problems 10‒16 years later,^6^ but changes in organizational justice or sleep were not analysed. A recent systematic review on the association between work environment factors and future sleep identified three relatively different studies on organizational justice, which in combination supported an association between high organizational justice and a lower risk of sleep disturbances.^4^ In all primary studies of the meta-analysis, justice referred mainly to the relational component of the organizational justice, i.e. fair treatment (after a reduction in pay),^9^ fairness of immediate superior’s leadership^10^ and relational justice.^6^ Thus, our findings are consistent with findings from this meta-analysis.^4^ They do not, however, support a cross-sectional study that showed an association between procedural injustice and insomnia symptoms.^8^

There are several potential reasons why relational justice could have more consistent associations with insomnia symptoms than procedural justice. Relational justice is, by definition, linked to the personal and proximal aspects of fair and just treatment by the supervisor, e.g. lack of kindness and consideration, whereas procedural injustice involves more general, distant decision-making procedures in the organization. Relational justice could also be linked to perceived social support, which is associated with sleep quality.^4^ In addition, procedural justice may change more slowly and through different processes compared with relational justice, and thus the effects on sleep might be less evident.

The mechanisms underlying the association between relational justice and insomnia symptoms are unknown. A plausible mechanism could be related to individual responses to the work environment. Thus, perceived injustice could induce the experience of psychological stress, including psychological and physical symptoms such as insomnia. Indeed, psychological stress is related to the onset or maintenance of insomnia symptoms.^4^^,^^5^ Moreover, sleep tends to improve after old age retirement,^21^ and this could be related to the elimination of work-related psychological stress. Depression and anxiety are additional plausible mechanisms that underlie the injustice-insomnia association: potentially consequences of injustice and causes of insomnia. However, depression and anxiety can also represent confounders that affect the perceptions of organizational justice. The latter is an unlikely source of bias in our study because including depression and trait anxiety as baseline covariates in the analysis had little effect on the associations. Further research on the mechanisms underlying the associations of relational justice and insomnia are needed, as the links between insomnia, work-related stress and mental health are complex.^22^

The observed association was robust to several pertinent confounding risk factors, such as sociodemographic factors, shift work, obesity, poor health behaviours, mental and physical health and sleep apnoea,^4^^,^^23^^,^^24^ highlighting the potential importance of perceived injustice for insomnia symptoms. Robust associations were also expected, given the nature of the exposure, the multistage study design and the baseline exclusions. More specifically, changes in organizational justice are more likely to result from factors related to the workplace than those related to the participant. Homogenization of the cohort in terms of baseline organizational justice further reduced variation in potential confounding factors.

This study also has some limitations. First, the numbers of participants were notably smaller in the favourable changes analysis than those in the analysis of unfavourable changes. This was due to our inclusion criteria; frequent insomnia symptoms and injustice at baseline are less common than good sleep and justice. Although the numbers differed, the results in both analyses were in the expected direction and robust. Second, we were only able to include self-reported measures of insomnia symptoms, although this is common in large-scale epidemiological studies^25^ and unlikely to distort the association, as the onset of insomnia was temporally distinguished from the unfavourable change in organizational justice. Furthermore, the insomnia symptom measure is not specific to clinical insomnia, and we only requested symptoms during the previous 4 weeks. Thus, we cannot determine with certainty whether the insomnia symptoms fluctuated or remained stable between the surveys. However, insomnia symptoms tend to be persistent and increase with age.^26^^,^^27^ Third, we also used self-reported data on work exposures. Although the association could be different for objective measures, these lack the components of perceived fairness and justice. In addition, as it is likely that people are not treated in the same way by their supervisors, even in the same work units, any aggregate measures are likely to produce different results and capture something other than justice perception. Having self-reported data on both exposure and outcome may cause concern regarding common method bias, i.e. those who report several symptoms also tend to report high exposure. As we addressed changes in exposure within the same individual in this study, this type of bias should be less of a concern.

Fourth, we were only able to include public sector employees, and this might limit the generalizability of the results to the private sector, which has a more equal sex distribution and different types of jobs. However, there is no particular reason to assume that the effects of justice on sleep should differ according to the employment sector, although the level of justice between organizations may differ. The cohort consisted mainly of women, and if women are more sensitive to social and relational issues than men, having a female-dominated cohort may have affected the results. However, since no sex interactions were found, this is unlikely to be a major issue. As the participants were of Caucasian ethnicity and from a Nordic welfare state, generalizability to other populations may be limited.

## Conclusions and implications

The results of this study suggest temporal associations between relational justice and sleep quality, an important determinant of employee well-being and working capacity. Further interventional research is needed to examine whether improvements in relational justice via, for example, training supervisors, improve sleep quality among employees. An intervention study has suggested that training managers to treat employees in an interactionally fair manner and with increased empathy and engagement is associated with beneficial effects on sleep among employees.^28^ As an unfavourable change in relational justice appears to increase the risk of insomnia symptoms, early detection and identification of psychosocial exposures at work may be important for the prevention of these problems.

## Supplementary Data

Supplementary data are available at *IJE* online.

## Funding

The work was supported by the Academy of Finland [grant numbers #287488, #294096, #286294, #294154, #267727, #258598, 265174 #292824 and #265977], and the participating organizations. S.S. was also supported by the Ministry of Education and Culture. M.K. was supported by Nordforsk, the Nordic Programme on Health and Welfare, the Finnish Work Environment Fund and the UK Medical Research Council (K013351). T.L. is guarantor of the present study.

**Conflict of interest:** None declared.

## Supplementary Material

Supplementary Data
