# Supplementary material for: Change in organizational justice as a predictor of insomnia symptoms: longitudinal study analysing observational data as a non-randomized pseudo-trial
Source: Int J Epidemiol. 2017 Jan 8;46(4):1277–84. doi: 10.1093/ije/dyw293 (PMC5837615; doi:10.1093/ije/dyw293)
Supplement: Supplementary Data [file dyw293_supp_ije-2016-05-0647-file006.docx]

**Online Appendices 1-4**

**Change in organizational justice as a predictor of insomnia symptoms: longitudinal study analyzing observational data as non-randomized pseudo-trial**

Tea Lallukka, Jaana I Halonen, Børge Sivertsen, Jaana Pentti, Sari Stenholm, Marianna Virtanen, Paula Salo, Tuula Oksanen, Marko Elovainio, Jussi Vahtera, Mika Kivimäki

**eTable 1.** Characteristics (numbers, %) of participants in the two included nested cohorts beginning in 2000‒2002 (Trial 1) and 2004 (Trial 2). Finnish Public Sector Study participants 2000‒2012.

|  | Trial 1 **(n=18432)** | | Trial 2 **(n=17757)** | |
| --- | --- | --- | --- | --- |
| **Sex** | N | % | N | % |
| Men | 3225 | 17.5 | 3145 | 17.7 |
| Women | 15207 | 82.5 | 14612 | 82.3 |
|  |  |  |  |  |
| **Married** |  |  |  |  |
| Single | 4102 | 22.5 | 3970 | 22.5 |
| Living without a partner | 14145 | 77.5 | 13650 | 77.5 |
|  |  |  |  |  |
| **Education** |  |  |  |  |
| Low | 1717 | 9.3 | 1067 | 6.0 |
| Intermediate | 6563 | 35.6 | 6225 | 35.1 |
| High | 10152 | 55.1 | 10465 | 58.9 |
|  |  |  |  |  |
| **Current smoker** |  |  |  |  |
| No | 14835 | 82.8 | 14508 | 83.5 |
| Yes | 3074 | 17.2 | 2878 | 16.6 |
|  |  |  |  |  |
| **Physically inactive** |  |  |  |  |
| No | 13867 | 76.0 | 13640 | 77.4 |
| Yes | 4377 | 24.0 | 3991 | 22.6 |
|  |  |  |  |  |
| **Heavy drinker** |  |  |  |  |
| No | 16819 | 91.7 | 16109 | 91.1 |
| Yes | 1517 | 8.3 | 1579 | 8.9 |
|  |  |  |  |  |
| **Body mass index** |  |  |  |  |
| Normal weight | 10427 | 57.9 | 9488 | 54.8 |
| Overweight | 5609 | 31.1 | 5631 | 32.5 |
| Obese | 1988 | 11.0 | 2210 | 12.8 |
|  |  |  |  |  |
| **Comorbid condition(s)** |  |  |  |  |
| No | 13226 | 71.8 | 12405 | 69.9 |
| Yes | 5206 | 28.2 | 5352 | 30.1 |
|  |  |  |  |  |
| **Shift work** |  |  |  |  |
| No | 12330 | 66.9 | 12038 | 67.8 |
| Yes | 6102 | 33.1 | 5719 | 32.2 |
|  |  |  |  |  |
| **Apnea** |  |  |  |  |
| No | 18228 | 98.9 | 17601 | 99.1 |
| Yes | 204 | 1.1 | 156 | 0.9 |

**eTable 2.** Associations between unfavorable change in organizational justice and subsequent insomnia symptoms (n=6307, eTable 2a), and between favorable change in organizational injustice and repeated insomnia symptoms (n=2903, eTable 2b) among Finnish public sector employees.

**a) Unfavorable change analysis**

|  | Model 1 |  |  | Model 2 |  |  |  |
| --- | --- | --- | --- | --- | --- | --- | --- |
|  | OR | 95% CI | | OR | 95% CI | |  |
| Organizational justice |  |  |  |  |  |  |  |
| remained high (reference) | 1.00 |  |  | 1.00 |  |  |  |
| decreased to low | 1.14 | 1.01 | 1.29 | 1.15 | 1.01 | 1.30 |  |
| Model 1 = crude model adjusted for trial number | | | | | | | |
| Model 2 = fully adjusted model adjusted simultaneously for trial number, sex, age, marital status, education, shift work, smoking, heavy alcohol use, low physical activity, body mass index, sleep apnea, and comorbid conditions (physician-diagnosed asthma, chronic obstructive pulmonary disease, hypertension, diabetes, and depression) | | | | | | | |

**b) Favorable change analysis**

|  | Model 1 |  |  | Model 2 |  |  |  |
| --- | --- | --- | --- | --- | --- | --- | --- |
|  | OR | 95% CI | | OR | 95% CI | |  |
| Organizational justice |  |  |  |  |  |  |  |
| remained low (reference) | 1.00 |  |  | 1.00 |  |  |  |
| increased to high | 0.84 | 0.72 | 0.98 | 0.85 | 0.73 | 0.99 |  |
| Model 1 = crude model adjusted for trial number | | | | | | | |
| Model 2 = fully adjusted model adjusted simultaneously for trial number, sex, age, marital status, education, shift work, smoking, heavy alcohol use, low physical activity, body mass index, sleep apnea, and comorbid conditions (physician-diagnosed asthma, chronic obstructive pulmonary disease, hypertension, diabetes, and depression) | | | | | | | |

**eTable 3.** Associations between unfavorable change in organizational justice and subsequent insomnia symptoms (n=6307, eTable 3a), and between favorable change in organizational injustice and repeated insomnia symptoms (n=2903, eTable 3b) without adjusting for depression among Finnish public sector employees.

**a) Unfavorable change analysis ***

|  | OR | 95% CI | |
| --- | --- | --- | --- |
| Relational justice |  |  |  |
| remained high (reference) | 1.00 |  |  |
| decreased to low | 1.16 | 1.03 | 1.30 |
|  |  |  |  |
| Procedural justice |  |  |  |
| remained high (reference) | 1.00 |  |  |
| decreased to low | 1.08 | 0.95 | 1.23 |
|  |  |  |  |
| Overall justice |  |  |  |
| remained high(reference) | 1.00 |  |  |
| decreased to low | 1.15 | 1.02 | 1.30 |

* Fully adjusted model adjusted simultaneously for trial number, sex, age, marital status, education, shift work, smoking, heavy alcohol use, low physical activity, body mass index, sleep apnea, and comorbid conditions (physician-diagnosed asthma, chronic obstructive pulmonary disease, hypertension, and diabetes)

**b) Favorable change analysis ***

|  | OR | 95% CI | |
| --- | --- | --- | --- |
| Relational justice |  |  |  |
| remained low (reference) | 1.00 |  |  |
| increased to high | 0.82 | 0.71 | 0.96 |
|  |  |  |  |
| Procedural justice |  |  |  |
| remained low (reference) | 1.00 |  |  |
| increased to high | 0.90 | 0.77 | 1.06 |
|  |  |  |  |
| Overall justice |  |  |  |
| remained low (reference) | 1.00 |  |  |
| increased to high | 0.85 | 0.72 | 0.99 |

* Fully adjusted model adjusted simultaneously for trial number, sex, age, marital status, education, shift work, smoking, heavy alcohol use, low physical activity, body mass index, sleep apnea, and comorbid conditions (physician-diagnosed asthma, chronic obstructive pulmonary disease, hypertension, and diabetes)

**eTable 4** Associations between unfavorable change in organizational justice and subsequent insomnia symptoms (n=6307, eTable 3a), and between favorable change in organizational injustice and repeated insomnia symptoms (n=2903, eTable 3b) additionally adjusting for trait anxiety among Finnish public sector employees.

**a) Unfavorable change analysis ***

|  | OR | 95% CI | |
| --- | --- | --- | --- |
| Relational justice |  |  |  |
| remained high (reference) | 1.00 |  |  |
| decreased to low | 1.14 | 1.01 | 1.29 |
|  |  |  |  |
| Procedural justice |  |  |  |
| remained high (reference) | 1.00 |  |  |
| decreased to low | 1.07 | 0.94 | 1.22 |
|  |  |  |  |
| Overall justice |  |  |  |
| remained high (reference) | 1.00 |  |  |
| decreased to low | 1.13 | 1.00 | 1.28 |

* Fully adjusted model adjusted simultaneously for trial number, sex, age, marital status, education, shift work, smoking, heavy alcohol use, low physical activity, body mass index, sleep apnea, and comorbid conditions (physician-diagnosed asthma, chronic obstructive pulmonary disease, hypertension, diabetes, and trait anxiety, score more than 2)

**b) Favorable change analysis ***

|  | OR | 95% CI | |
| --- | --- | --- | --- |
| Relational justice |  |  |  |
| remained low (reference) | 1.00 |  |  |
| increased to high | 0.84 | 0.72 | 0.98 |
|  |  |  |  |
| Procedural justice |  |  |  |
| remained low (reference) | 1.00 |  |  |
| increased to high | 0.92 | 0.78 | 1.08 |
|  |  |  |  |
| Overall justice |  |  |  |
| remained low (reference) | 1.00 |  |  |
| increased to high | 0.86 | 0.73 | 1.00 |

* Fully adjusted model adjusted simultaneously for trial number, sex, age, marital status, education, shift work, smoking, heavy alcohol use, low physical activity, body mass index, sleep apnea, and comorbid conditions (physician-diagnosed asthma, chronic obstructive pulmonary disease, hypertension, diabetes, and trait anxiety, score more than 2)
